# Retraction Note: Pedal to the metal: accelerating intracerebral hemorrhage treatment with robotic-assisted surgery. A systematic review & meta-analysis of clinical effectiveness

**DOI:** 10.1007/s10143-026-04248-3

**Published:** 2026-03-26

**Authors:** Paweł Łajczak, Anna Łajczak

**Affiliations:** Department of Biophysics, Faculty of Medical Sciences in Zabrze, Medical University of Silesia in Katowice, Jordana 18, Zabrze, 40-043 Poland

**Retraction Note: Neurosurgical Review (2024) 47:799**

10.1007/s10143-024-03039-y

The Editor-in-Chief has retracted this article. An investigation by the Publisher found that a number of articles, including this one, were submitted over a short space of time and show strong indications that the text was generated by a large language model (LLM) without proper disclosure by the authors. These articles are, therefore, in breach of the Journal's editorial policy and are being retracted.

Paweł Łajczak on behalf of both authors disagrees with the retraction.

